# A systematic and quantitative method for wound-dressing evaluation

**DOI:** 10.1186/s41038-015-0013-9

**Published:** 2015-09-03

**Authors:** Xiaorong Zhang, Rui Xu, Xiaohong Hu, Gaoxing Luo, Jun Wu, Weifeng He

**Affiliations:** Chongqing Key Laboratory for Disease Proteomics, State Key Lab of Trauma, Burns and Combined Injury, Institute of Burn Research, Southwest Hospital, The Third Military Medical University, Chongqing, 400038 China

**Keywords:** Wound dressing, Evaluation, Wound model

## Abstract

**Background:**

For patients with skin defects such as burns, wound dressing plays important roles in protecting the wound. Before a novel wound dressing is applied to a patient, a series of tests should be performed to ensure its safety and efficacy. Different types of animal wound-healing models have been used to study the bio-function of different wound dressings; however, a systematic way to evaluate the effect of a wound dressing on wound healing and cutaneous regeneration is lacking.

**Methods:**

In the study presented here, full-thickness wound models were established in mice, and a systematic way to quantitatively analyze the wound-healing process and the histological results is described.

**Results:**

It was found that the rate of wound healing in the tested wound dressing (TWD) group was higher than that in the control group, and the re-epithelialization and the formation of granulation tissue were enhanced when the TWD was applied. Meanwhile, the inflammatory response was attenuated in the TWD group, and more mature and better aligned collagen fibers in the healed wound tissue was found in the TWD group compared with that in the control group.

**Conclusions:**

A systematic, quantitative way to analyze the effect of a wound dressing on wound healing was established. And it might be helpful for the design of wound dressing in the future.

## Background

As the largest organ in the human body, the skin plays important roles in continuously maintaining human life. Due to direct exposure to thermal, mechanical, and chemical damage, skin defects such as burns, ulcers, and other skin loss may easily happen [1]. Wounds, especially extensive full-thickness wounds, cannot heal spontaneously. Therefore, a wound dressing is needed to cover these wounds.

An ideal wound dressing may possess many features to protect the wound and to reconstruct the physical barrier in the wound. For instance, superior mechanical properties are important for protecting the wound from mechanical damage; a satisfactory water vapor transmission rate and fluid uptake ability can provide a moist microenvironment to accelerate wound healing [2, 3]. Favorable histocompatibility is also an important feature to guarantee safety when the wound dressing is applied to the wound. Overall, an ideal wound dressing should protect against contamination, outside disturbances, and a loss of elements from the body. At the same time, the dressing should supply a suitable microenvironment for wound healing or wound bed preparation.

Before a new wound dressing is permitted to be tested in a clinical trial, it is very important to investigate the wound-healing process when the wound dressing is applied in an animal model. So far, several different types of animal models have been suggested [4–6]. For instance, a full-thickness skin defect Bama pig model was used to study a new type of dermal equivalent [7], a circular full-thickness wound model in Sprague-Dawley rats was used to evaluate a chitin film compared with gauze [8], and a full-thickness round-wound model in mice was used to investigate a physiologically active polysaccharide hydrogel [9]. However, studies often aim to study the structure, physical properties, safety, and histocompatibility of wound dressings; a systematic, detailed, and precise evaluation of the bio-function of wound dressings in the wound healing is lacking in most of these studies.

The purpose of the present study was to provide a systematic way to evaluate the effect of wound dressings on wound healing. Two types of full-thickness wound models in mice were used to study wound dressings, and a way to quantitatively analyze the wound-healing process and the histological results was established.

## Methods

### Animals

BALB/c mice (male, 18–20 g) were purchased from the Experimental Animal Department of the Third Military Medical University. All of the animal protocols were approved by the Institutional Animal Care and Use Committee of the Third Military Medical University. The animals were individually housed in plastic cages and were fed sterile rodent chow and water ad libitum. The mice were allowed to acclimatize for 7 days before the experiments.

### Reagents and equipment

Paraformaldehyde (Sigma, cat. no. 158127, USA), pentobarbital sodium salt (Sigma, cat. no. 57-33-0, USA), hair removal cream (Veet, cat. no. 20070747, France), a digital camera (SONY, DSC-T100, Japan), an optical microscope (CTR6000, Leica, Germany), and Image-Pro Plus 6.0 (IPP 6.0) software (Media Cybernetics, USA) were used.

### Wound models and wound analysis

#### Anesthesia and depilation

BALB/c mice were placed under general anesthesia with 1 % pentobarbital via intraperitoneal injection at a dose of 5–8 μl/g. The hair on the dorsal part of each mouse was removed 1–2 days before surgery. By using hair removal cream, it is easy to remove the hair without any damage to the mouse’s skin.

#### Mouse full-thickness wound surgery

The dorsal part of each mouse was disinfected with iodophors, followed by a rinse with 75 % (*v/v*) ethanol. Two types of wound models were used here: a two-wound model created using a sterile biopsy punch, with a diameter of each wound of 0.4 cm, and a larger one-wound model created on the back of the mouse with sterile scissors and tweezers, with a wound size of 1 cm × 1 cm.

A sterile round marker (diameter 0.4 cm) or a square marker (area 1 cm^2^) was placed next to the wound, which was immediately photographed using a digital camera. The marker was used to measure the wound area, and the photograph was taken vertical to the wound and the marker to avoid bias.

The test wound dressing (TWD) was sutured and dressed on the wound with a 6.0 nylon. The TWD used in this study was a patented microporous membrane, and a wound without any treatment served as a control.

### Critical step

The control group is very important for wound-dressing studies and should be appropriately chosen based on your own experimental design. For example, traditional wounds treated with Vaseline gauze or other types of wound dressings or wounds without any treatment can be used as the control group.

### Measurement of the wound area

At day 3 and day 7 post-surgery, the wound dressing was removed, and the wound was photographed. Based on the picture, the area of the wound was measured using IPP 6.0 software. Briefly, the margin of each wound and the marker were carefully traced, and the number of pixels encompassing each wound and marker was calculated. As the area of the marker was known, the number of pixels corresponding with the wound was then converted into square centimeters, after which the rate of wound healing could be calculated as follows:

$$ \mathrm{Rate}\ \mathrm{of}\ \mathrm{wound}\ \mathrm{healing} = \left(\mathrm{A}\mathrm{W}\mathrm{i} - \mathrm{AW}\mathrm{n}\right)\ /\ \mathrm{A}\mathrm{W}\mathrm{i} \times 100\ \% $$

where AWi represents the area of the initial wound and AWn represents the remaining area of the wound on the nth day post-surgery [10, 11]. Five mice in each group were used to determine the approximate time of wound closure.

### Pause point

The days on which to uncover the wound dressing and to take photographs of each wound are chosen based on the experimental design; we prefer to perform these steps at day 3 and day 7.

### Histological observation

#### Hematoxylin and eosin (HE) staining and analysis

At day 3 and day 7, after the wound was photographed, the mice were sacrificed, and the histology of the wound sections was then investigated. The wound tissue, with adjacent normal skin, was carefully biopsied fixed in 4 % formaldehyde and then embedded in paraffin and sectioned at a thickness of 5 μm. The sections were stained with HE for histological analysis.

The granulation thickness and the length of the epithelial tongue (newly generated epidermis) were measured with IPP 6.0 software. The length of the epithelial tongue was defined as the distance between the advancing edges of the epidermal keratinocytes and the hair follicles in non-wounded skin [12, 13].

The number of inflammatory cells in the dermis was determined in five random fields. The inflammatory cells were automatically identified, counted, and highlighted by the IPP 6.0 software, according to the morphology of the cells. In addition, the inflammatory cells could be counted manually.

### Masson’s trichrome staining and analysis

The mice were sacrificed 3 days after complete re-epithelialization. Re-epithelialized tissues from the site of initial injury were obtained and fixed in 4 % formaldehyde. The fixed specimens were sectioned and stained with Masson’s trichrome stain. The staining was performed as follows: each section was deparaffinized, hydrated, stained with iron hematoxylin, and rinsed with deionized water. The sections were then stained with Biebrich scarlet-acid fuchsin. Subsequently, the sections were counterstained with aniline blue.

The thickness of the epidermis was measured using IPP 6.0 software.

Moreover, to measure the density of the collagen fibers, as previously described [14–16], the areas of the collagen fibers and of the total section were measured by counting the pixels in the images. The density of the collagen fibers was defined as follows: area of collagen staining / total area of section × 100 %.

### Statistical analysis

Statistical analysis was performed using Statistical Package for the Social Sciences (SPSS) version 18.0 (SPSS GmbH, Munich, Germany). In this study, all data are presented as the mean ± standard deviation (SD). The significance of differences between two data sets was tested with unpaired *t* tests. Differences were considered significant when *P* < 0.05.

## Results

### The full-thickness wound models were established in mice

**Fig. 1:**
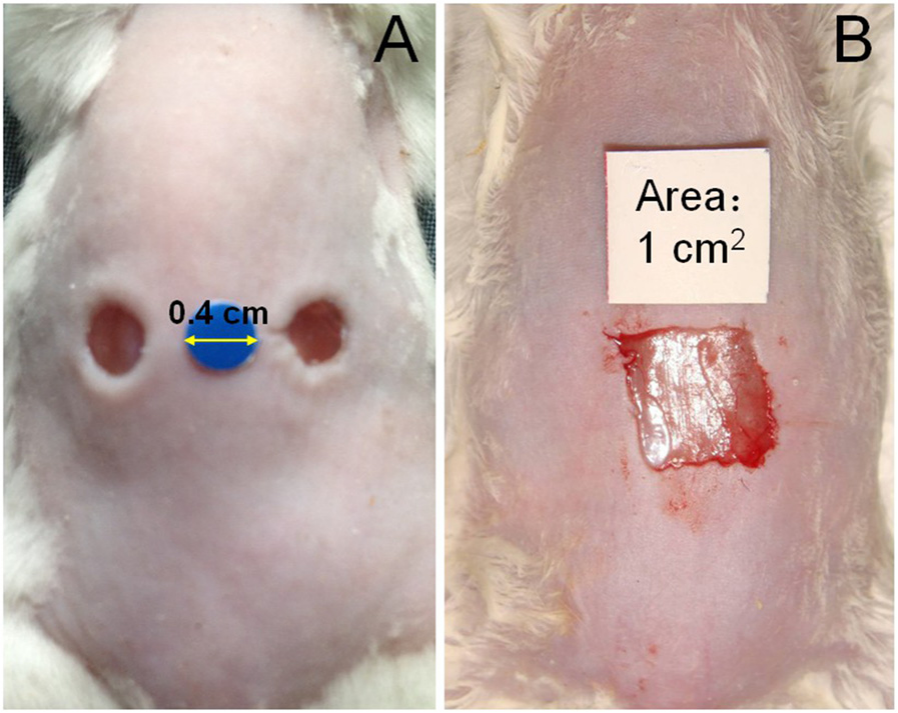
Mouse full-thickness wound model. **a** The two-wound round-wound model. The diameter of the wound was 0.4 cm. **b** The one-wound square-wound model. The area of the wound was approximately 1 cm^2^

A two-wound round-wound model and a one-wound square-wound model were established using our methods (Fig. 1). Each wound was generated beyond the panniculus carnosus (full-thickness wounds), and the area of each wound was determined using IPP 6.0 software.

### Gross observation

#### The macroscopic appearance of the wounds

**Fig. 2:**
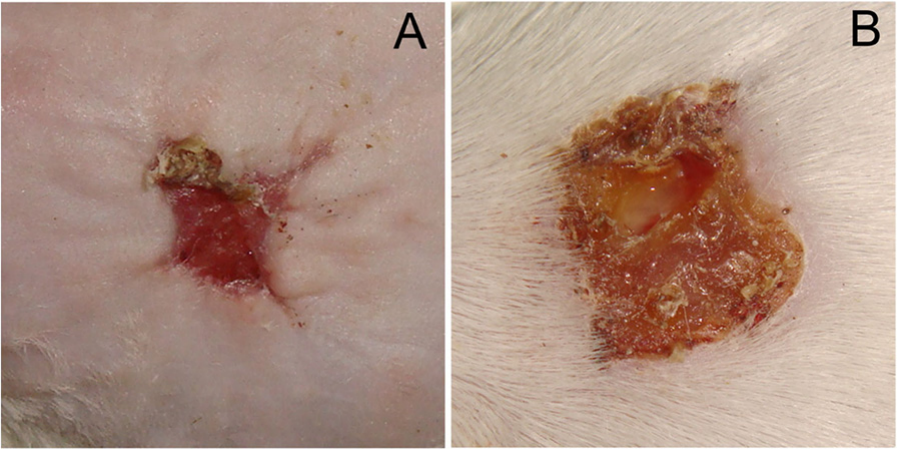
Macroscopic appearance of the wound in the **a** TWD group and **b** control group. In the TWD group, it was found that the wound was moist and clean, and newly formed epidermis could be observed at the wound margin. In contrast, in the control group, a scab formed, and inflammatory exudates could be found

Based on the photographs at different times post-surgery, the macroscopic appearances of the wounds covered with different wound dressings were different. A wound that was moist and clean, without too much exudate (Fig. 2a), was conducive to wound healing.

#### The rate of wound healing and the wound-closure time

**Fig. 3:**
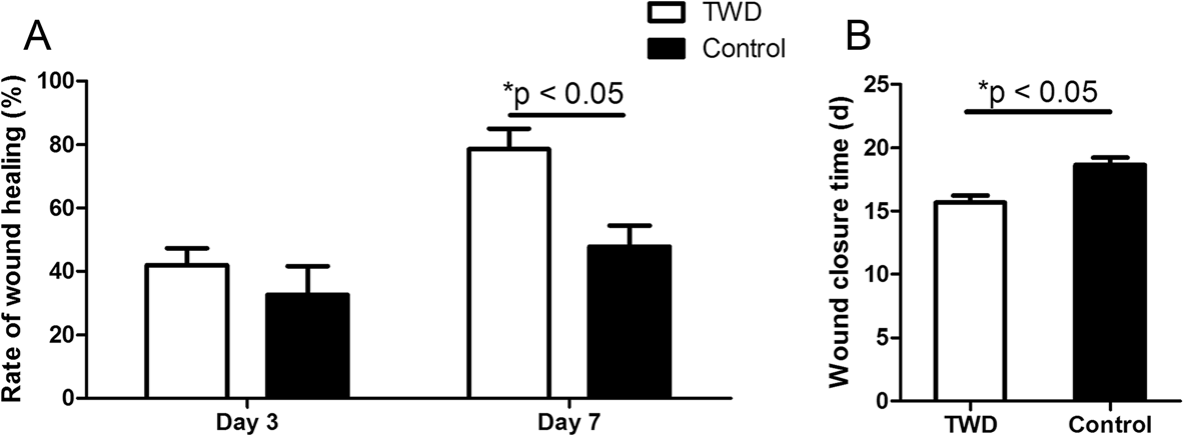
The rate of wound healing (**a**) and time of wound closure (**b**). It was found that the rate of wound healing in the TWD group was higher than that in the control group, and application of the TWD significantly shortened the wound-closure time. The values are the mean ± standard deviation (SD) (*n* = 5), *P < 0.05

The wound areas at day 3 and day 7 were measured for the different groups, and the rate of wound healing was calculated. At day 7, it was found that the average rate of wound healing in the TWD group was 78.7 %, which was higher than that in the control group. Moreover, the wound-closure times of the different groups were determined, and the wound-closure time in the TWD group was shorter than that in the control group (Fig. 3).

### Histological results and analysis

#### HE staining and analysis

##### Granulation tissue thickness

**Fig. 4:**
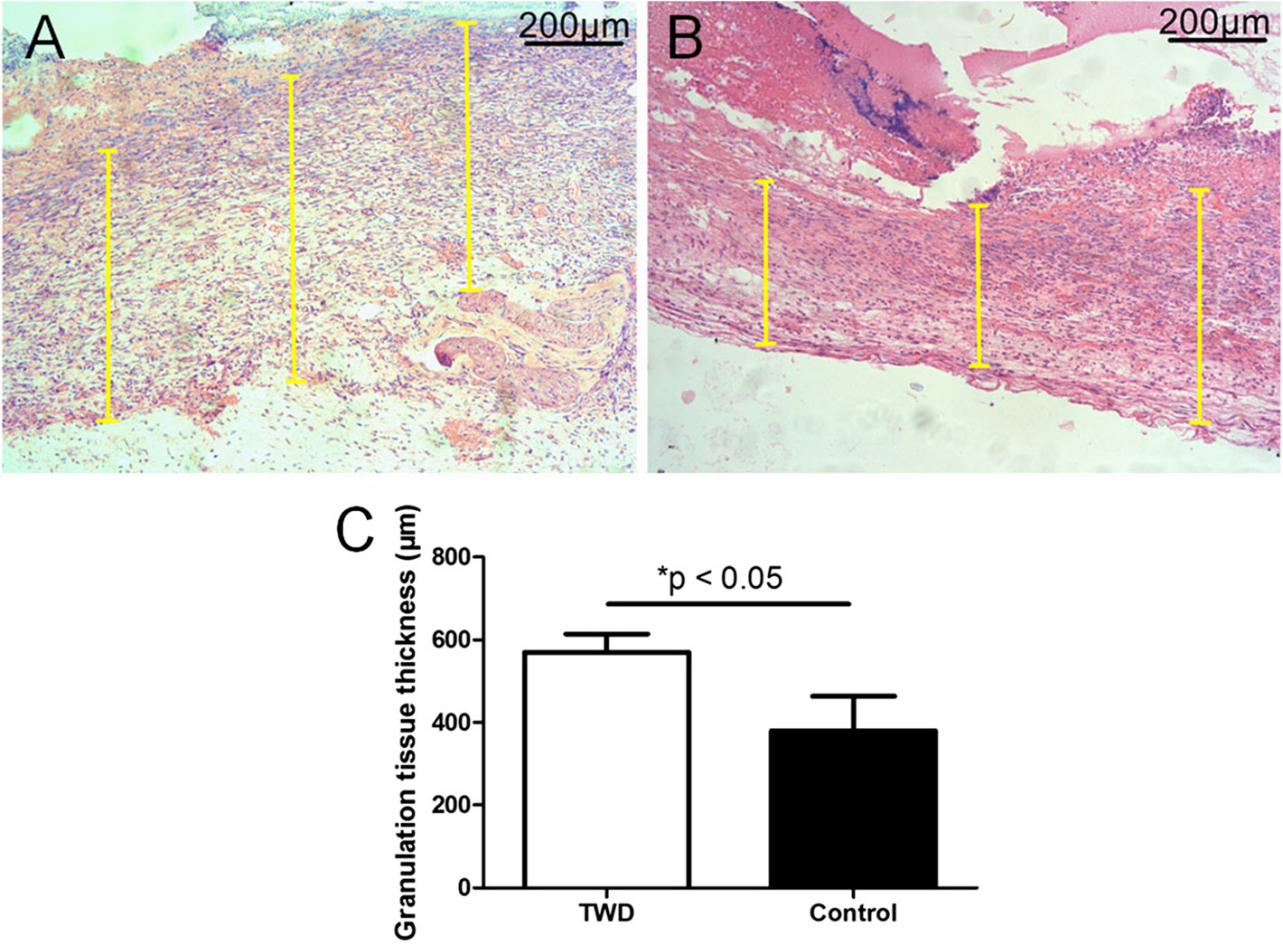
Measurement of the granulation tissue thickness at day 7 in the **a** TWD group and **b** control group. **c** Granulation tissue formation was enhanced when the wounds were covered with the TWD. The values are the mean ± standard deviation (SD) (*n* = 5), *P < 0.05

Granulation tissue thicknesses were measured in the different groups (Fig. 4) based on the histological sections. It was found that the granulation tissue thickness in the TWD group (570.6 ± 43.9 μm) was greater than that in the control group (379.4 ± 84.1 μm) at day 7, as shown in Fig. 4.

##### The length of the newly generated epidermis

**Fig. 5:**
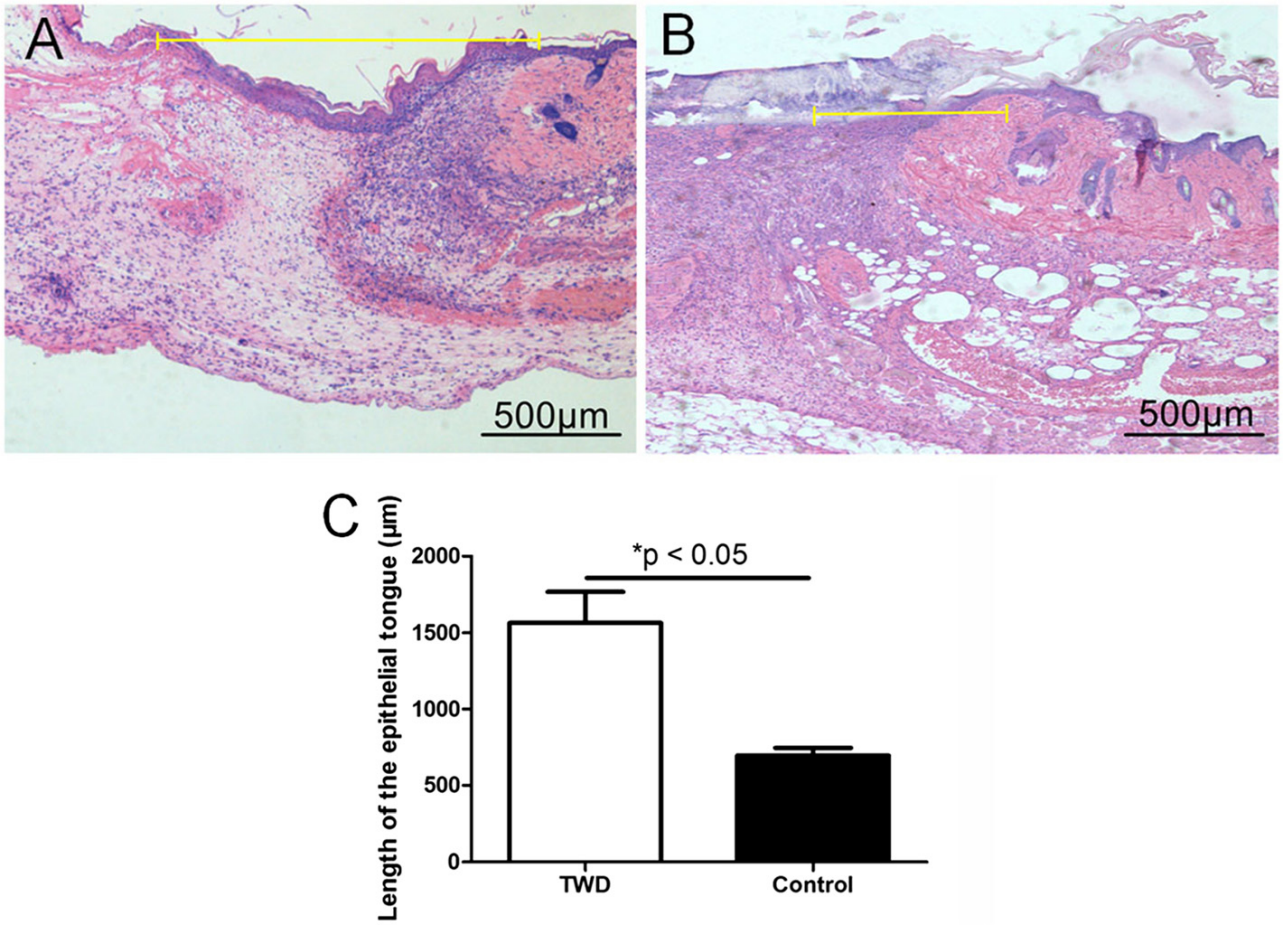
Measurement of the length of the newly generated epidermis in the wounds at day 7 in the **a** TWD group and **b** control group. **c** It was found that the length of the newly generated epidermis in the TWD group was longer than that in the control group. The values are the mean ± standard deviation (SD) (*n* = 5), *P < 0.05

The length of the newly generated epidermis was measured with IPP 6.0 software (Fig. 5). At day 7, it was found that the length of the newly generated epidermis in the TWD group (1566.0 ± 203.0 μm) was longer than that in the control group (695.9 ± 50.3 μm).

##### The inflammatory response in the wound tissue

**Fig. 6:**
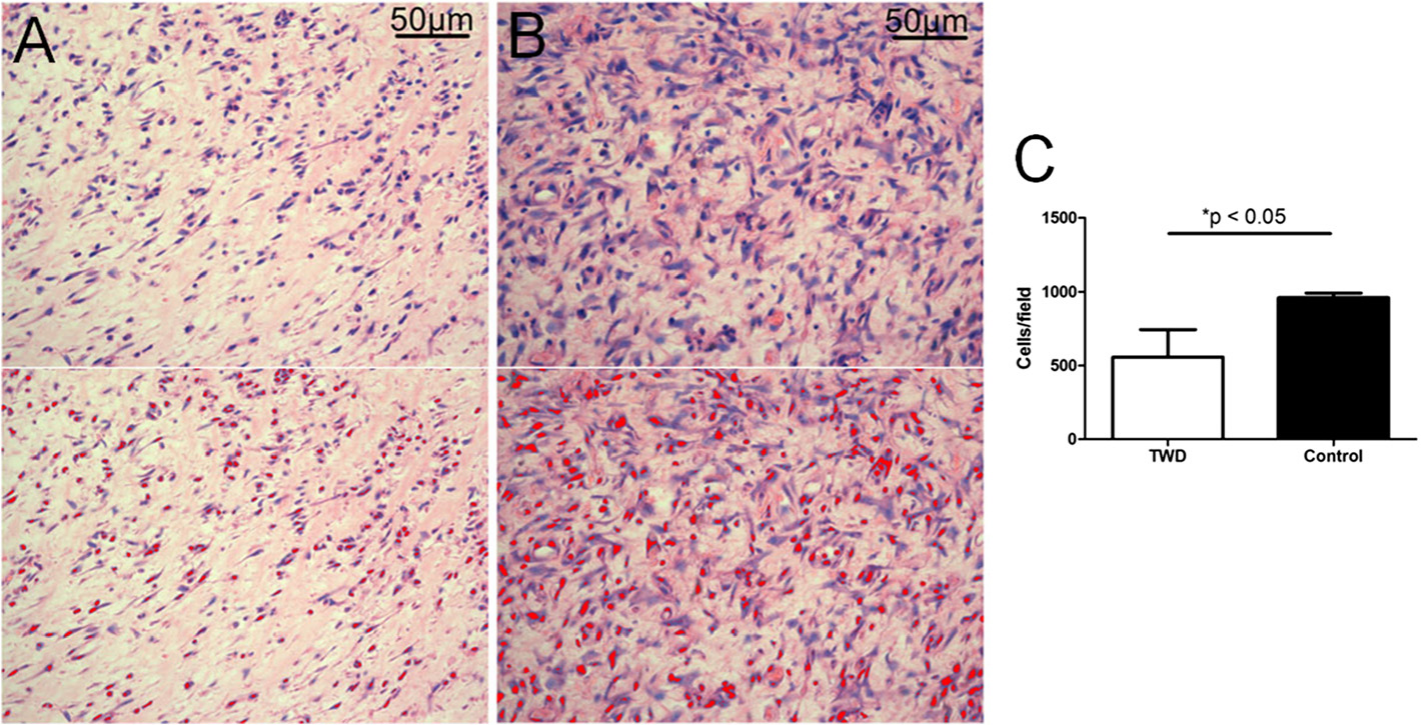
The number of inflammatory cells was counted to evaluate the inflammatory response in the wounds of the **a** TWD group and **b** control group at day 7; inflammatory cells were highlighted (*lower panel*) using IPP 6.0 software. **c** More inflammatory cells were found in the dermis in the control group than in the TWD group. The values are the mean ± standard deviation (SD) (*n* = 5), *P < 0.05

The number of inflammatory cells was determined to evaluate the inflammatory response in the wounds. At day 7 post-surgery, a significant difference in the number of inflammatory cells was found between the two groups (Fig. 6, TWD bar vs. control bar, *P* < 0.05).

#### Masson’s trichrome staining and analysis

##### The thickness of the newly re-epithelialized epidermis

**Fig. 7:**
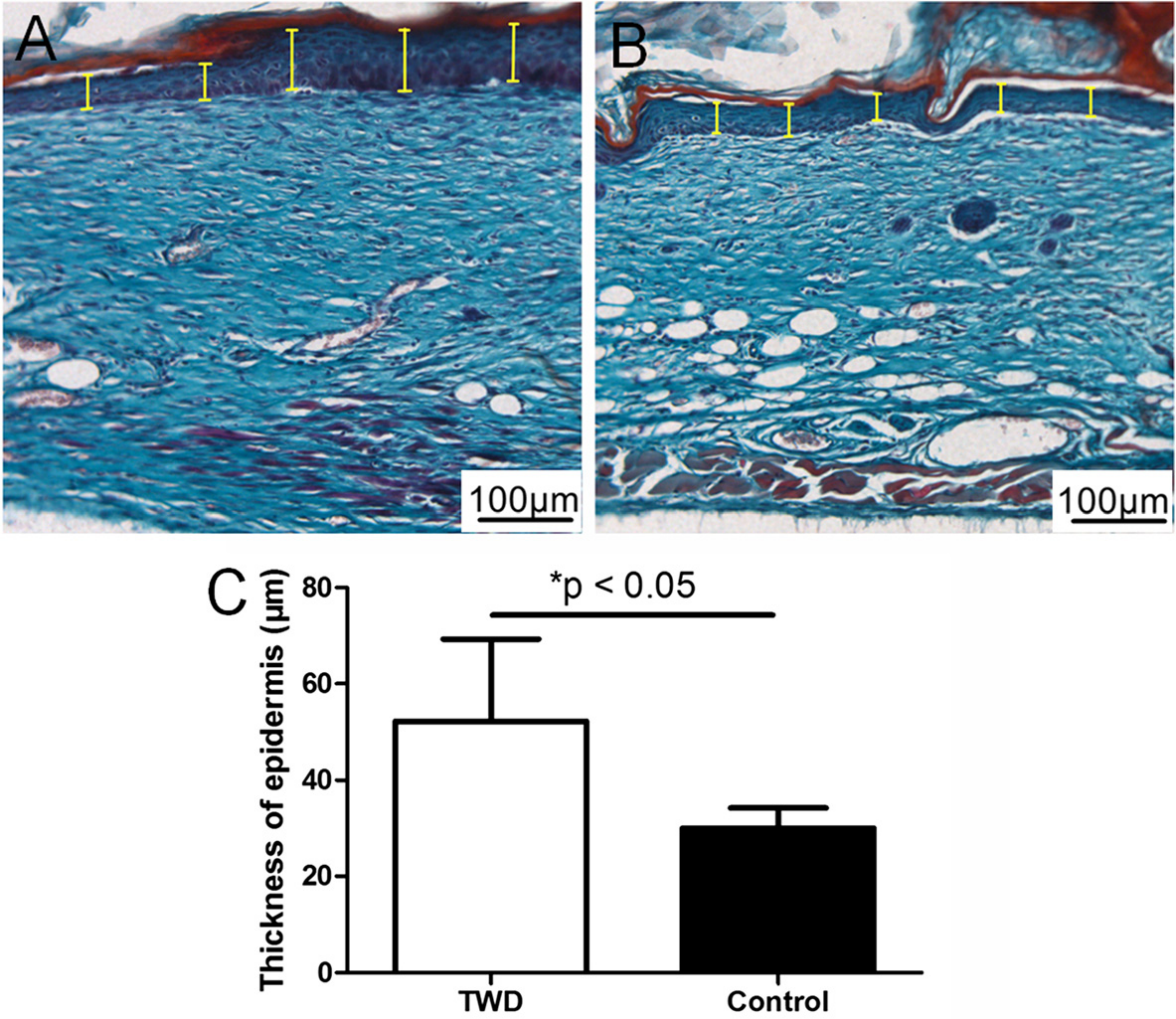
Measurement of the thickness of the newly generated epidermis in the healed wounds in the **a** TWD group and **b** control group. **c** Application of the TWD significantly increased the thickness of the newly generated epidermis. The values are the mean ± standard deviation (SD) (*n* = 5), *P < 0.05

To further evaluate the quality of healing, Masson’s trichrome staining was performed to observe the newly re-epithelialized epidermis, and the thickness of the newly re-epithelialized epidermis was measured. As shown in Fig. 7, the average thicknesses of the newly formed epidermis in the TWD group and control group were 52.3 and 30.0 μm, respectively, which were significantly different.

##### Collagen density

**Fig. 8:**
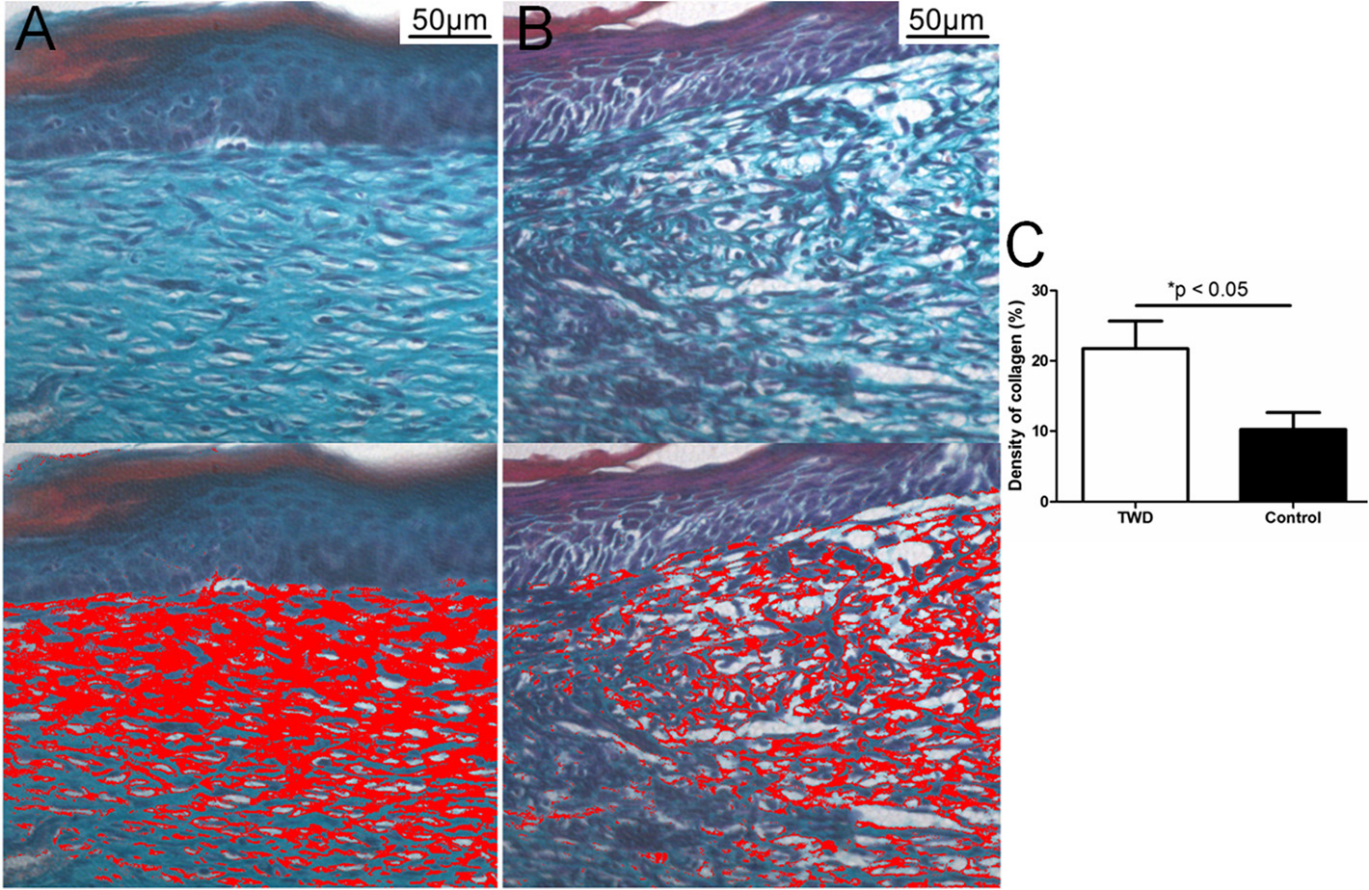
Masson’s trichrome staining of the healed tissue in the **a** TWD group and **b** control group; collagen fibers were highlighted (*lower panel*) using IPP 6.0 software. **c** The density of collagen fibers in the TWD group was increased compared with that in the control group. The values are the mean ± standard deviation (SD) (*n* = 5), *P < 0.05

Masson’s trichrome staining was performed to stain the collagen fibers in the healed wound tissue. Moreover, to quantitative analyze the amount of collagen fibers, the collagen densities in the healed wound tissues covered with different wound dressings were measured. As shown in Fig. 8, the density of the collagen fibers in the TWD group was 21.7 %, which was significantly increased compared with the density in the control group (10.2 %).

## Discussion

When a new type of wound dressing is prepared, it is very important to investigate the wound-healing process when the wound is exposed to the wound dressing in an animal model. However, a systematic, detailed and precise evaluation of the bio-function of wound dressings in the wound healing is lacking in most published studies. Therefore, here, we presented a systematic, quantitative way to analyze the effect of a wound dressing on wound healing.

In the present study, full-thickness wound models were used for the evaluation of the TWD in mice. It is known that for animals with loose skin, such as a mouse or a rat, the wound-healing process is not exactly the same as for tight-skinned species, such as humans or pigs [17]. Wound contraction is a key parameter for wound closure in mice; however, it is not notable in human wounds, in which re-epithelialization is considered more important than wound contraction. The skin structure and wound-healing mechanism of the mouse are different from those of humans, which is the limitation of mouse wound models. However, we believe that a mouse model is still a useful research tool for preclinical study, and it is relatively low in cost and easy to use compared with pig and other models. In addition, mouse models have been widely used for wound-dressing evaluation or wound-healing research [4, 9, 10, 13].

Therefore, here, we presented two types of wound models, i.e., a two-wound round-wound model and a one-wound square-wound model (Fig. 1). The wound model should be appropriately chosen based on your own experimental design. The two-wound model is much better for controlling for biological variation because the TWD group and control group can be studied within one mouse, which is always better than a control from a different animal. However, when we try to evaluate more than three types of wound dressings at one time, it is impossible to create so many wounds on one mouse. In this case, the one-wound model can be used. Additionally, a large wound makes it easy for us to observe the wound-healing process and to find differences among different groups. To control for biological variation when the one-wound model is used, the mice should be of the same sex and should have similar weights and hair follicle cycles. In addition, the mice should be randomly assigned to the different groups.

As a moist environment is conducive to wound healing [18–20], wounds that were moist and clean, without too much exudate, were beneficial to wound healing (Fig. 2a). In contrast, a red and swollen wound with a scab or with extensive inflammatory exudate might hamper wound healing (Fig. 2b). By observing the wounds treated with different wound dressings and analyzing the rates of wound healing in the different groups, the best dressing could be identified.

Granulation tissue is very important for wound healing because it fills the defect, prevents bacterial invasion, and prepares the matrix for cell proliferation and migration. Re-epithelialization is also an important factor for wound closure. Therefore, the thickness of the granulation tissue and the length of the epithelial tongue were measured based on the sections in our study. The thickness of the granulation tissue and the length of the epithelial tongue in the TWD group were increased compared with those in the control group, which indicated that the TWD was conducive to wound healing (Figs. 4 and 5).

Modulation of the inflammatory response is also an important factor during the wound-healing process. It has been reported that androgens could reduce the inflammatory response, thus resulting in accelerated wound healing [21]. Hence, the attenuated inflammatory response in the wound tissue in the TWD group further supported the hypothesis that the corresponding wound dressing was conducive to wound healing (Fig. 6). In our research, neutrophils, lymphocytes, and macrophages, among other cells, were all automatically identified as inflammatory cells, counted and highlighted by IPP 6.0 software, according to the cell morphology. In this way, this software saves time when we have to examine dozens of histopathological pictures. However, the limitation of this method is that cells without typical morphology may be identified incorrectly. Thus, if we want to identify and count the cells in a precise way, we prefer to do it manually.

As newly formed epidermis is fragile, without the support of the mature dermal matrix, the thickness of the newly formed epidermis is important for protecting the healed area. A comparatively thicker epidermis might indicate a better skin barrier, and the corresponding wound dressing might be better than the others (Fig. 7).

Collagen is the main component of the dermal matrix. Compared with the control group, the more mature and better aligned collagen fibers in the healed wound tissue indicated that a better quality of healing was established when the TWD was applied (Fig. 8) [1]. Here, we should note that although measurement of the density of collagen fibers based on histopathological pictures has been widely used in many studies [14–16], there are many other ways that are more precise for collagen assessment, such as Western blotting or the determination of hydroxyproline expression.

## Conclusions

In the study, a systematic, quantitative way to analyze the effect of a wound dressing on wound healing was established. By analyzing the wound-healing process and the histological results in the way presented here, a new wound dressing could be evaluated using an animal model before it was permitted to be tested in a clinical trial. Our study might be helpful for the design of wound dressing in the future.

## Abbreviations

AWi: area of the initial wound
AWn: remaining area of the wound on the nth day post-surgery
HE: hematoxylin and eosin
IPP 6.0: Image-Pro Plus 6.0
SD: standard deviation
SPSS: Statistical Package for the Social Sciences
TWD: test wound dressing
